# Biodegradation of Ammonium Ions and Formate During Ammonium Formate Metabolism by *Yarrowia lipolytica and Pichia guilliermondii* in a Batch Reactor

**DOI:** 10.1007/s11270-018-3795-0

**Published:** 2018-05-05

**Authors:** M. N. Nsoe, G. P. Kofa, K. S. Ndi, B. Mohammadou, M. Heran, G. J. Kayem

**Affiliations:** 1 Institute of Technology, University of Ngaoundere, P.O. Box 455, Ngaoundere, Cameroon; 2 Water Treatment and Filtration Research (Chem. Eng.) Group, Department of Process Engineering, ENSAI, University of Ngaoundere, P.O. Box 455, Ngaoundere, Cameroon; 3 The Higher Institute of the Sahel (ISS), University of Maroua, P.O. Box 46, Maroua, Cameroon; 4 Institute Europeen of Membrane UMR 5635 (CNRS-ENSCM-UMII), 005 – 34095, Montpellier CED, Place Eugène Bataillon France

**Keywords:** Ammonium formate, Rubber wastewater, Batch reactor, *Yarrowia lipolytica*, *Pichia guilliermondii*, Inhibitor substrat

## Abstract

The use of microorganisms for the biodegradation of pollutants is increasingly being studied. But at high concentrations, these pollutants become rather inhibitors for the metabolism of microorganisms. In this study, the biodegradation of ammonium formate at various concentrations (1.59–7.94 mM) by *Yarrowia Lipolytica* and *Pichia guilliermondii* isolated from the rubber effluent is studied by following the variation of ammonium ions and formate. A fitting of eight models of substrate inhibition was performed and the parameters were determined by nonlinear regression using MATLAB 8.5 ©. The *R*^2^ and the RSME allow to choose the best model. The results show that ammonium ions (3.17 mM ammonium formate) are used as substrate; no inhibition is observed. But beyond this concentration, the inhibition effect begins to be observed with the specific rates of ammonium biodegradation which decrease. Formate monitoring reveals that is used as the main source of energy and does not inhibit the growth of yeasts. The models of Luong and Webb seem to be more appropriate for predicting the observed phenomena of inhibition. For *Yarrowia lipolytica*, *R*^2^ = 0.958 and 0.998 with RSME = 0.005342 and 0.003433, for *Pichia guillermondii*, *R*^2^ = 0.999 and 0.992 with RSME = 0.0005121 and 0.001212.

## Introduction

Rubber production is in a full rise all over the world. It moved from 7 to 11 million tons in 2014 (LMC Rubber 2013). The harvested latex is usually stabilized by ammonia and then transported to the factory where it is coagulated by the addition of formic acid. Rubber industry produces large volume of wastewater which needs to be treated prior disposal in order to avoid malodorous problem and pollution effects in the receiving water. During the coagulation process, the reaction between ammonia and formic acid produces ammonium formate, which is the major component of wastewater and accounts for 23.4% (Atagana et al. 1998). The ammonium formate is toxic and the risks of exposure vary from one concentration to another; transient and reversible effects on health are observed from 3.7 μg L^−1^.41 μg L^−1^, these effects become harmful and irreversible (severe irritations and permanent damage to the eyes, irritation of the mucous membranes and respiratory tract, stomach pain, difficulty breathing, and finally gastrointestinal irritation, abdominal pain). Above 240 μg L^−1^, it leads to cell death (EPA USA 2016). The consumption of water containing the ammonium ion leads to the synthesis of carcinogenic nitrosamines, the formation of methaemoglobin in infants (Sulaiman et al. 2010; Arimoro 2009; Bougard 2004) and the inhibition of certain Pathways of the digestive and respiratory tract. At the environmental level, eutrophication of mangroves and rivers and the death of biological (Nsoe et al. 2016, Tekasakul 2010, Seneviratne 1997) are observed. The increase in ammonium ion concentrations in the environment leads in the long term to an imbalance of the nitrogen cycle and an acceleration of denitrification which can increase the concentrations of nitric oxide and nitrous oxide responsible for the destruction of the layer ozone. As for the formate ion, the reduction produces the CO_2_. Although its concentration in air is well below the toxicity threshold, emissions are not directly harmful to humans. On the other hand, CO_2_ emissions are mainly responsible for global warming, which can cause major health and safety problems for living organism (natural disasters, diseases, displacement of populations). This makes this compound the second most responsible for more than 39% of the destruction of the ozone layer. Given the health and environmental issues associated with ammonium formate, the need to eliminate this pollutant in these effluents before their release into the environment becomes an imperative. Sridhar et al. (2014), Peitz (2012), and Dobrovolná and Ervený (2000) proposed to fight ammonium formate by catalysis process. But by-products of this process (HCONH_2_, HCN, NH_3_) still represent a source of pollution for the environment. Moreover, the large volumes of waste water produced by these industries cannot be treated by this process which consumes a lot of energy. Biological treatments are increasingly explored for the treatment of these effluents (Ndi et al. 2016; Pillai and Girish 2014; Senthil et al. 2012; Swarna Smitha et al. 2012; Shruthi et al. 2012; Cheria and Jayachandran 2009). However, low purifying efficiencies have always been observed, mostly due to variations in the concentration of pollutants (NH_3_) which become inhibitors even at low concentration (Vadivelu et al. 2007). In this case, mathematical modeling is useful for understanding the behavior of biological processes and predicting the concentrations of components in the system (Dutta et al. 2015; Tazda et al. 2013) to avoid inhibition and increase treatment efficiency. In this study, two strains of yeast (*Yarrowia lipolytica* and *Pichia guilliermondii*) were isolated from rubber effluents. The degradation of ammonium formate was monitored as well as the potential for inhibition of this molecule. The inhibitory effects of nitrogen on yeast were assessed and the experimental data were in accordance with the substrate inhibition model.

## Materials and Methods

### Yeast Inoculum

The yeast strains used in this work were isolated from the effluents of a rubber industry thanks to Nsoe protocol (Nsoe et al. 2013). The yeast characteristics are shown in Table 1.

**Table 1:** Characteristics of yeast strains

Code	Yeast color	Zeta potential pH (5.7–7)	Yeast shape	Yeast size (μm)	Growth in selective media	
					Acid medium	Alkaline medium
*Yarrowia lipolytica* (LR)	Red	− 25.3 to − 37.8	Ovoid	0.3–150	+	+
*Pichia guilliermondii* (LB)	White	− 27.8 to − 30.5	Ovoid	0.2–100	+	+

Yeast were cultivated on the following growing medium (Atagana et al. 1998): meat extract 1 g L^−1^ (Sherlan réf 07-075, Spain), yeast extract 2 g L^−1^ (OXOID code L21, England), peptone 5 g L^−1^ (Liofilchem réf 610038, Italy), and sodium chloride 5 g L^−1^ (Jeulin réf 107 115, France) at pH = 6.5.

### Synthetic Influent

The synthetic influent was built to ensure usually concentration found in rubber factories. Ammonium formate was the only source of carbon and nitrogen. It was added in order to cover different nitrogen concentrations: from 1.59 to 7.94 mM (22.3 to 111 mg N L^−1^). Ammonium formate were mixed to mineral salt medium (in g L^−1^): MgSO_4_ (0.2), CaCl (0.02), KH_2_PO_4_ (60), K_2_HPO_4_ (14). A stock solution of microelements (in g L^−1^)—ZnSO4 (10.90), FeSO4 (5), MnSO4 (1.54), and CuSO4 (0.39)—was prepared and added to the influent at 0.1% (*V*/*V*). Then, 0.25 g L^−1^ of chloramphenicol was added to inhibit the bacterial growth and the synthetic influent was sterilized at 110 °C for 10 min.

### Experimental Set-up: Batch Bioreactor

**Fig. 1:**
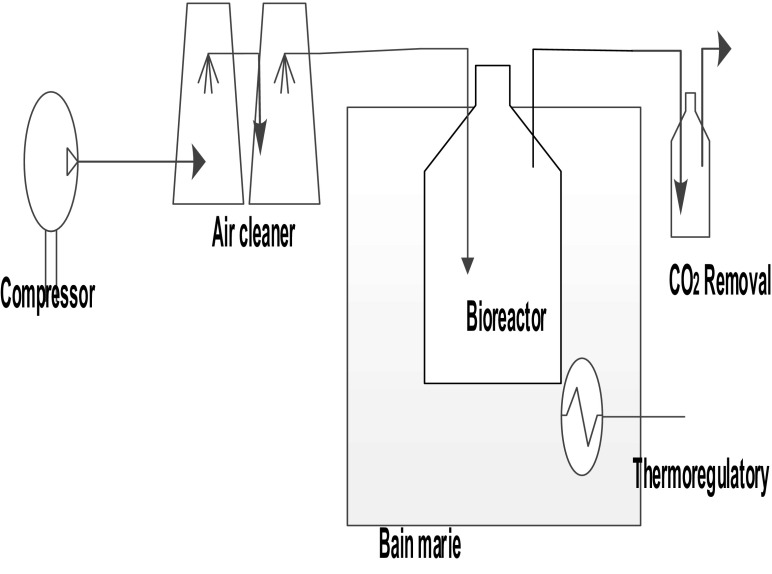
Batch bioreactor

Figure 1 shows us the Batch Bioreactor mounted for this experiment, it consists of several bioreactors of 1 L (with a useful volume of 0.8 L) were used and immersed in a water bath to maintain the temperature at 25 °C. Aeration was supplied thanks to an air compressor (GIANESSIEDILIO, NML 58629, LT 100, ATE 12TEMPA, Italy) to ensure that dissolved O2 was not a limiting factor for biomass respiration and growth. In order to reduce bacterial contamination and CO_2_ content, air inlet undergoes a succession of washing solution with 1 mM sodium hydroxide and 1 mM hydrochloric acid. Then, air outlet was connected to a 500-mL jar containing a 0.5 mM sodium hydroxide solution in order to quantify the CO_2_ released during the catabolism of ammonium formate.

### Analytical Methods

Samples were centrifuged at 1200 rpm, 4 °C, and 10 min. in 5000*g* a Kendro-Heraeus PrimoR centrifuge (Biofuge, Germany). Then, ammonium ions (NH_4_^+^) where titrated by a portable mini-photometer of mark HANNA Checker HC ® HI 733 Woonsocket RIUSE ROMANIE. The formate is determined by the method of the center of expertise in environmental analysis of Quebec code: MA.405-C11, 2014).

### Determination of Maximum Enzymatic Activity

In order to establish the maximum enzymatic activity curve as a function of the substrate concentrations (formate and ammonium), the slope of the biodegradation kinetics curves of the substrate (formate and ammonium) as a function of time allows us to determine the enzyme activity for each strain at each substrate concentration.

### Calibration and Validation of Inhibition Model by Ammonium Formate

A large set of different widely published substrate inhibition models were used to analyze experimental data (Table 2). The parameters of different models were estimated from the experimental results using MATLAB v.7.1. Since the models had nonlinear coefficients, the parameters were estimated iteratively with nonlinear least square algorithm.

**Table 2:** Models of inhibition by the substrate (Dutta et al. 2015; Tazda et al. 2013; Agarry et al. 2010; Amrouche et al. 2010; Agarry and Solomon 2008)

Model	Equations
Andrews	$\mu =\frac{\mu_{\mathrm{max}}\cdot {\gamma}_s}{\left({K}_s+{\gamma}_s\right)\left(1+\frac{\gamma_s}{K_I}\right)}$ (1)
Luong	$\mu =\frac{\mu_{\mathrm{max}}\cdot {\gamma}_s}{\left({K}_s+{\gamma}_s\right)}{\left[1-\frac{\gamma_s}{\gamma_s^{\ast }}\right]}^n$ (2)
Han–Levenspiel	$\mu =\left[{\left(1-\frac{\gamma_s}{\gamma_s^{\ast }}\right)}^n\right]\frac{\gamma_sC}{\gamma_s+{K}_M\left[{\left(1-\frac{\gamma_s}{\gamma_s^{\ast }}\right)}^m\right]}$ (3)
Haldane	$\mu =\frac{\mu_{\mathrm{max}}\cdot {\gamma}_s}{K_s+{\gamma}_s+\frac{\gamma_s^{\ast }}{K_I}}$ (4)
Moser	$\mu =\frac{\mu_{\mathrm{max}}\cdot {\gamma}_s^n}{\left({K}_s+{\gamma}_s^n\right)}$ (5)
Ailba	$\mu =\frac{\mu_{\mathrm{max}}\cdot {\gamma}_s}{\left({K}_s+{\gamma}_s\right)}\exp -\frac{\gamma_s}{K_I}$ (6)
Yano	$\mu =\frac{\mu_{\mathrm{max}}\cdot {\gamma}_s}{K_s+{\gamma}_s+\frac{\gamma_s^2}{K_I}+\left(1+\frac{\gamma_s}{K}\right)}$ (7)
Edward	$\mu ={\mu}_{\mathrm{max}}\cdot {\gamma}_s\left[\exp \left(\frac{-{\gamma}_s}{K_I}\right)-\exp \left(\frac{-{\gamma}_s}{K_I}\right)\right]$ (8)
Webb	$\mu =\frac{\mu_{\mathrm{max}}\cdot {\gamma}_s\left(1+\frac{\gamma_s}{K_I}\right)}{K_s+{\gamma}_s+\frac{\gamma_s^2}{K_I}}$ (9)

List of symbol: *K*_*I*_ : inhibition constant for ammonium (mM); *γ*_*s*_***:*** Initial cutin concentration(mM); *n*,*m*: constant parameters; *μ****:***specific growth rate, h^−1^; *μ*_max_ : maximum specific growth rate, h^−1^; *K*_*s*_: half-saturation constant for ammonium, (mM);${\gamma}_s^{\ast }:$ critical ammonium concentration (mM); *K* : Webb constant (mM); *C*: cell concentration in Han–Levenspiel model (mM)

## Results and Discussion

### Kinetics of Ammonium Biodegradation by Yeast Strains at Different Concentrations of Ammonium Formate

**Fig. 2:**
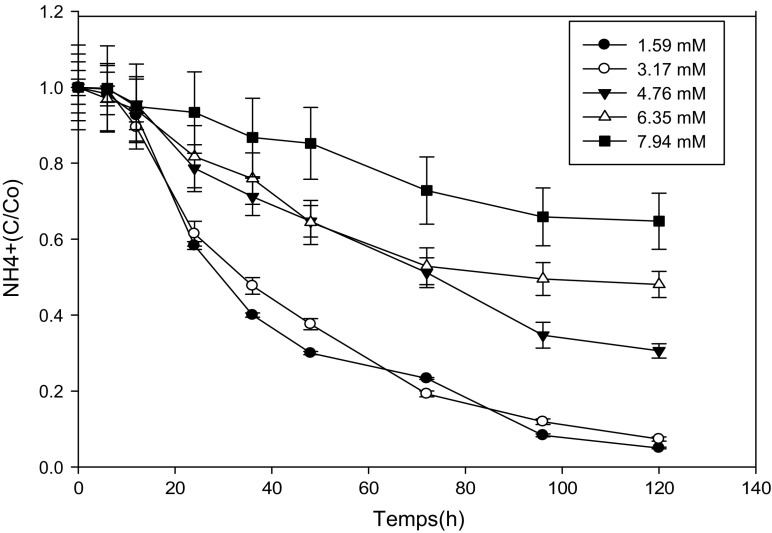
Variation of NH4^+^ as a function of time at different concentrations of ammonium formate for the *Yarrowia lipolytica* strain

**Fig. 3:**
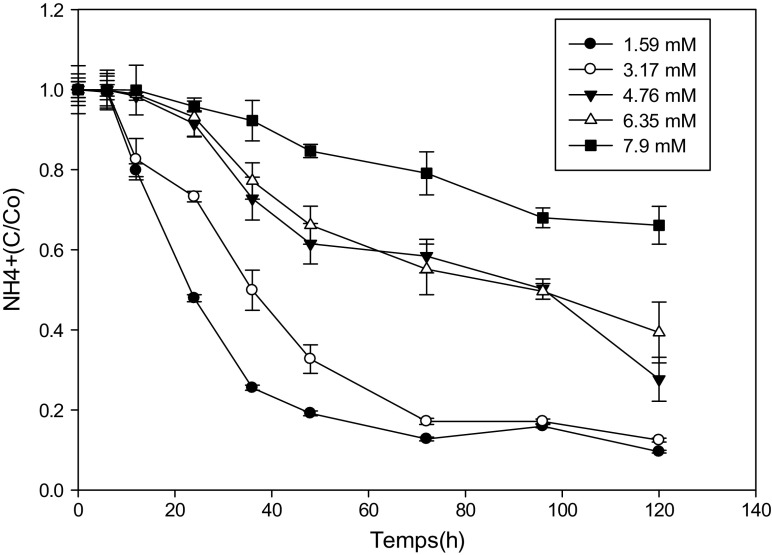
Variation of NH4^+^ as a function of time at different concentrations of ammonium formate for the *Pichia guilliermondii* strain

Figures 2 and 3 show the variation of the ammonium ions as a function of time for each concentration of ammonium formate. We find that yeasts assimilate ammonium ions regardless of the concentration of ammonium formate because all curves have a decreasing rate. But the percentage of abatement varies from one strain to another and from one concentration to another. In addition to all the concentrations of ammonium formates, there is a latency time which shows that there is either an adaptation time for the biodegradation of ammonium ions or that this source of energy would be a source of energy which is not directly used by yeasts. In contrary, the percentage of ammonium ions catabolized by yeasts is inversely proportional to the concentration of the ammonium ions present in the medium. What shows that it is the ammonium ions could inhibit yeast metabolism.

### Kinetics of Biodegradation of Total Organic Carbon by Yeast Strains at Different Concentrations of Ammonium Formate

**Fig. 4:**
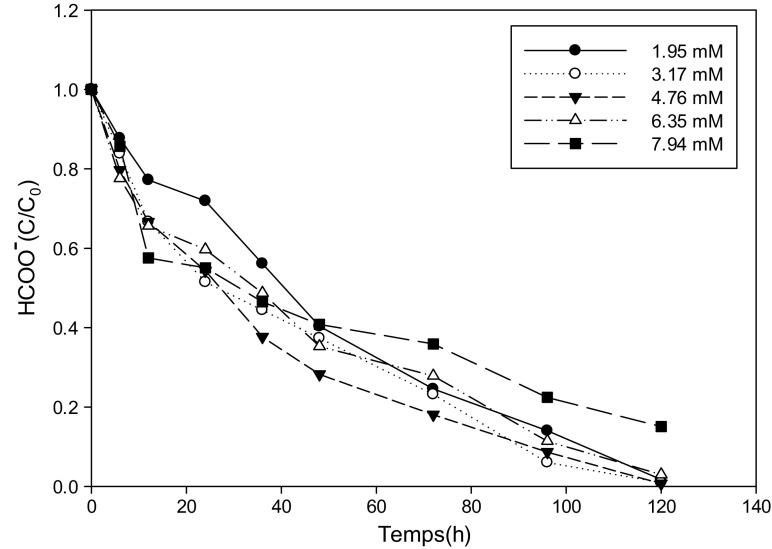
Variation of formate as a function of time at different concentrations of ammonium formate for the *Pichia guilliermondii* strain

**Fig. 5:**
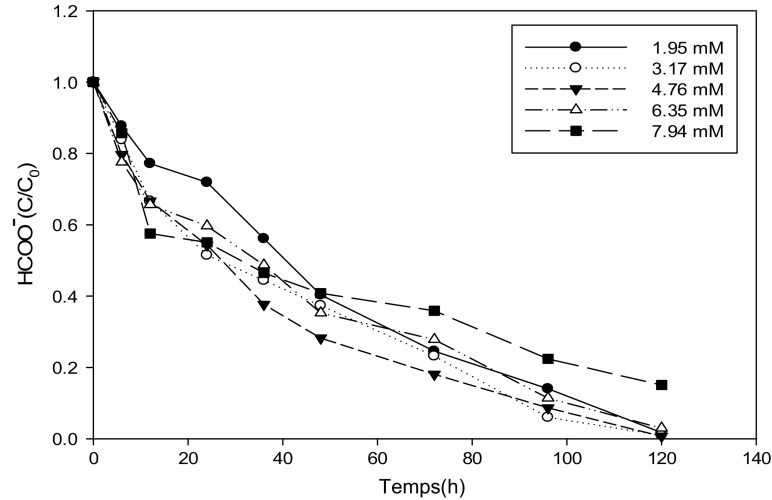
Variation of formate as a function of time at different concentrations of ammonium formate for the *Yarrowia lipolytica* strain

Figures 4 and 5 present the variation of formate as a function of time during the biodegradation of ammonium formate at different concentrations. All the figures present a decreasing pace whatever the strain without a latency time that might suggest that carbon is the main source of energy for growth yeasts compared to biodegradation curves of ammonium ions or there is a time of adaptation. We also find that for concentrations between 1.95 and 6.35 mM in ammonium formate, the percentage of abatement of formate is 98% regardless of the strain. The shape of the curves at 7.94 mM shows that this molecule is still being assimilated by yeasts and the percentage of abatement is lower.

### Influence of Ammonium Concentration Maximum Enzymatic Activity

**Fig. 6:**
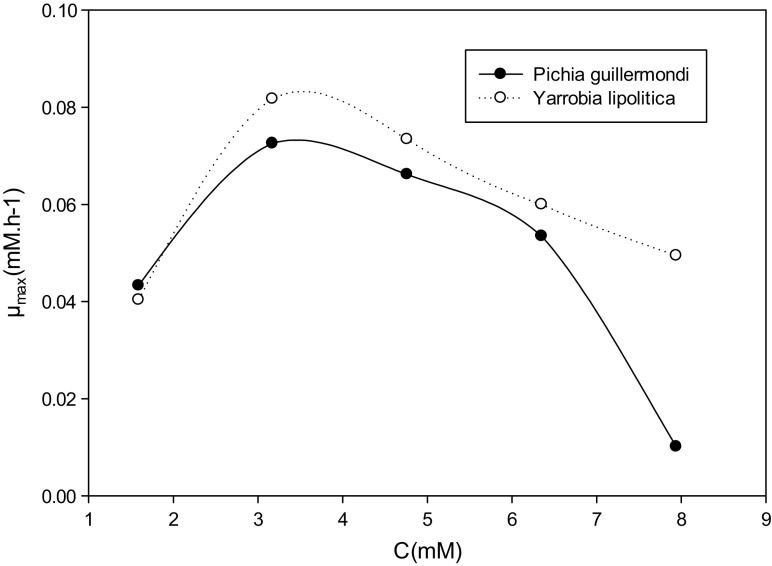
Influence of the initial ammonium concentration on the maximum enzymatic activity for *Yarrowia lipolytica and Pichia guilliermondii* strains

The maximum enzymatic activity as a function of the initial substrate concentration is shown in Fig. 6 for the two strains studied. These curves have a bell-like appearance and have two phases. A phase where the specific growth rates and maximal enzymatic activity increase with the formate concentration (1.59–3.17 mM) and a phase of decline of the specific growth rate and maximum enzymatic activity from 4.76 mM (Dutta et al. 2015; Agarry et al. 2010; Dey and Mukherjee 2010) proposed that this bell-like appearance at high substrate concentrations reveals inhibition by the substrate.

### Influence of the Formate Concentration on the Maximum Enzyme Activity

**Fig. 7:**
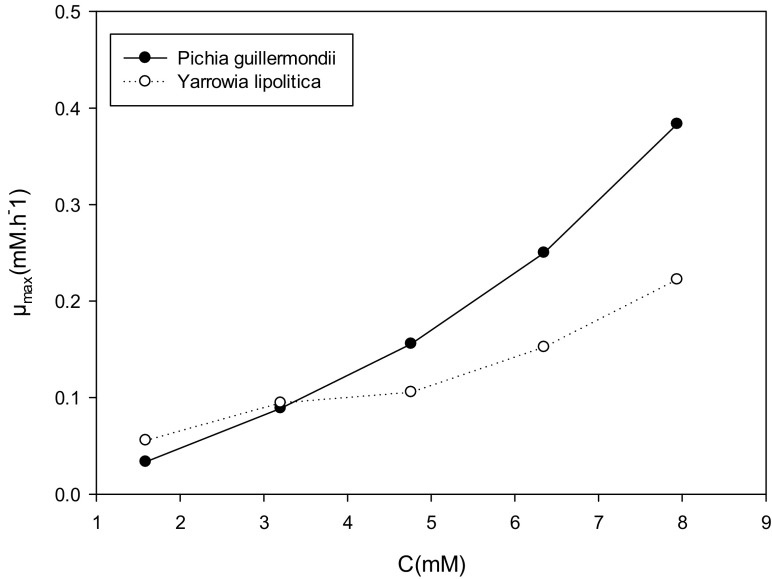
Influence of the initial formate concentration sur l’activité enzymatique maxiamle for *Yarrowia lipolytica and Pichia guilliermondii* strains

Figure 7 shows the variation of the maximum enzymatic activity as a function of the initial substrate concentration for the two strains studied. We find that the maximum enzyme activity increases with the concentration of formate. This suggests that formate is not responsible for inhibition but rather a primary source of energy. In addition, we find that up to 3 mM, both strains have the same maximum enzyme activity. But after this value, the enzymatic activity of *Pichia guilliermondii* becomes greater than that of *Yarrowia lipolytica* up to 8 mM where a doubling of this value is observed (0.4 μ_max_ h^−1^) for *Pichia guilliermondii* and (0.2 μ_max_ h^−1^) for *Yarrowia lipolytica.*

### Estimation of the Model and the Inhibition Parameters by Ammonium Formate

The biochemical parameters (*K*_I_, *K*_M_, *K*_S_, *μ*_max_) as well as the *R*^2^ and the RSME were estimated using the nonlinear regression of various models presented in Table 3 for the *Pichia guilliermondii* strain and Table 4 for the *Yarrowia lipolytica* strain. It is observed that the Luong model has the highest *R*^2^ (0.98), followed by the Webb model (*R*^2^ = 0.96), the Yano model (*R*^2^ = 0.91), and the Andrews model *R*^2^ = 0.91), show a good apparent adjustment between the experimental values and the model values for the *Pichia guilliermondii* strain. Thus, the square root of the mean square of the residuals (RMSE) between the experimental values and the models are respectively 0.0034, 0.0053, 0.0076, and 0.0055 for the four models. The *Yarrowia lipolytica* strain is the Luong model with the highest *R*^2^ (0.99), followed by the Webb model (*R*^2^ = 0.99), the Andrews model (*R*^2^ = 0.98), and finally the Yano model (*R*^2^ = 0.93) and the RMSEs are respectively 0.0012, 0.00051, 0.0026, and 0.0075. *K*_I_ values vary from strain to strain and from model to strain. The *K*_i_ models of Andrews, Aiba, Webb, Yano, Haldane, and Teisser are respectively 0.47, − 6396, − 8.54, 8.54, 0.20, and 6, 45 mM for *Pichia guilliermondii* and 1.17, − 1.02e 04, 0.09, 0.22, 0.37, and 7.04 mM for *Yarrowia lipolytica*. The *K*_I_ of the model of Aiba is weak which shows that with this model, the yeasts are insensitive to the inhibition. But for both strains, the *K*_I_ values of the Teisser model (4.76 and 5.91 mM) and Haldane (0.65 and 0.82) are substantially identical for both strains.

**Table 3:** Biochemical parameters after the fitting of the different models of inhibition by the substrate following the biodegradation of the ammonium ions for the *Pichia guilliermondii* strain

Models	*μ*_max_	*K*_S_	*K*_I_	${\gamma}_s^{\ast }$	*K*	*n*	*m*	*K*_m_	*R* ^2^	RMSE
Andrews	0.77	34.68	1.17	–	–	–	–	–	0.98	0.0026
Aiba	0.09	2.56	13	–	–		–	–	0.89	0.0068
Moser	0.24	0.25	–	–	–	0.29	–	–	0.76	0.010
Webb	0.26	0.89	0.094	–	0.91	–	–	–	0.99	0.00051
Luong	2.51	172.2		162.7		0.63	–	–	0.98	0.0012
Edward	0.02	0.95	0.22		0.26	–	–	–	0.93	0.0075
Haldane	0.09	0.65	0.37	50.71	–	–	–	–	0.89	0.0096
Teisser	0.44	4.76	7.04	–	–	–	–	–	0.92	0.0059
Han–Levenspiel	0.18	–	–	48.67	–	43	4	503.04	0.78	0.02

**Table 4:** Biochemical parameters after the fitting of the different models of inhibition by the substrate following the biodegradation of the ammonium ions for the strain *Yarrowia lipolytica*

Models	*μ*_max_	*K*_S_	*K*_I_	${\gamma}_s^{\ast }$	*K*	*n*	*m*	*K*_m_	*R* ^2^	RMSE
Andrews	1.31	71.14	0.47	–	–	–	–	–	0.91	0.0055
Aiba	0.07	2.24	6396				–	–	0.72	0.0096
Moser	4.83	4.96	–	–	–	0.36	–	–	0.60	0.011
Webb	0.98	4.09	8.24	–	4.11	–	–	–	0.95	0.0053
Edward	1.65	33.16		29.4		0.56	–	–	0.98	0.0034
Yano	0.0015	43.29	8.54		6.05	–	–	–	0.91	0.0076
Haldane	0.068	0.82	0.20	16.29	–	–	–	–	0.72	0.013
Teisser	1.586	5.91	6.45						0.87	0.0064
Han–Levenspiel	0.21	–	–	77.21	–	57	3	430	0.67	0.031

The maximum enzymatic activity (*μ*_max_) also varies from one strain to another and from one model to another. For *Pichia guilliermondii*, the highest value of *μ*_max_ is predicted by the Moser model (4.83 h^−1^) and the lowest by the Yano model (0.0015 h^−1^). The highest value of *μ*_max_ is predicted by the Luong model (2.51 h^−1^) and the lowest by the Yano model (0.021 h^−1^) for the *Yarrowia lipolytica* strain. Agarwal et al. (2009) modeling the degradation of insoluble cellulose found that the Han–Levenspiel model gives an ideal value. Agarry and Solomon in 2008 studied the degradation of phenol by the fluorescence of Pseudomonas and observed that the experimental data of *μ*_max_ corresponded well to the Haldane model. Dutta et al. in 2015 by studying the production of cutinase by *Pseudomonas cepacia* did not conclude on the ideal model. Tazda et al. 2013 found that the Yano and Andrews model best showed *μ*_max_ when biodegradation of malathion. The Luong and Haldane models predict the critical substrate concentration at which enzymatic activity is nothing. The critical concentrations for the Luong and Haldane models are 29.4 and 16.29 mM for *Yarrowia lipolytica* and 162.7 and 50.71 mM, respectively. The observation that emerges is that no model is standard to inhibitions by substrates. Different models could be applied to different systems.

## Conclusion

In this study, the biodegradation of ammonium formate by *Yarrowia lipolytica* and *Pichia guilliermondii* isolated from a rubber effluent was studied by following the variation of ammonium ions and formate in an aerated batch reactor. The results show that at 3.17 mM, ammonium ions are used as a substrate with an increase in maximum enzyme activity. But beyond this concentration, the maximum enzyme activity decreases. This is not the case for formate; the maximum enzymatic activity increases with the concentration of formate. The observation that emerges is that no model is standard to inhibitions by substrates. Different models could be applied to different systems. The models of Luong and Webb seem to be more appropriate for predicting the observed phenomena of inhibition. For *Yarrowia lipolytica*, *R*^2^ = 0.958 and 0.998 with RSME = 0.005342 and 0.003433, for *Pichia guilliermondii*, *R*^2^ = 0.999 and 0.992 with RSME = 0.0005121 and 0.001212.
